# Molecular remission using three monoclonal antibodies followed by allogeneic bone marrow transplantation in an infant with refractory ALL

**DOI:** 10.1007/s00277-020-03957-z

**Published:** 2020-02-18

**Authors:** Bernd Gruhn, Grit Brodt, Susan Wittig, Thomas Ernst, Jana Ernst

**Affiliations:** 1 Department of Pediatrics, Jena University Hospital, Jena, Germany; 2 Department of Internal Medicine II, Jena University Hospital, Jena, Germany

Dear Editor,

Acute lymphoblastic leukemia (ALL) in infants differs from ALL in older children [1] leading to a poor prognosis [2]. We present a 10-week-old infant diagnosed with immature ALL with myeloid markers in a foreign university hospital. After induction therapy according to LAL/SHOP protocol, 10% leukemic cells were detected in the bone marrow. Treatment was changed to fludarabine, cytarabine, and granulocyte colony-stimulating factor with doxorubicin. Afterwards, still 5% leukemic cells were detected in the bone marrow, so the bispecific T cell engager antibody blinatumomab was given. Due to increasing leukemic cells during the infusion, antibody therapy was stopped and clofarabine, cyclophosphamide, and etoposide were administered. Unfortunately, 31% leukemic cells were identified thereafter. Because of chemotherapy-refractory leukemia, a palliative oral treatment with mercaptopurine was started. However, palliative situation was not accepted by the parents and the infant was admitted to our hospital.

Genomic DNA was isolated from leukemic cells for molecular characterization. A MLL-MLLT3/AF9 rearrangement was detected and used as a marker for minimal residual disease (MRD). For further molecular characterization, targeted deep next-generation sequencing was performed for a panel of 54 leukemia-associated genes [3]. Interestingly, no mutation was found.

Since the CD33 antigen was strongly expressed in the first immunophenotyping, we administered the anti-CD33 monoclonal antibody gemtuzumab ozogamicin (GO) twice, two weeks apart (6 mg/m^2^), being well investigated in children with AML [4]. As CD38+ leukemic cells with loss of CD33 and CD22 were detected after GO infusion, the anti-CD38 antibody daratumumab was given alternatingly twice, two weeks apart (16 mg/kg). Daratumumab is established in multiple myeloma therapy [5] and showed promising results in preclinical examinations in T-ALL [6]. However, it has never been used in infants. Because reappearing leukemic cells were positive for CD22 and negative for CD33 and CD38 (Table 1), we administered a third antibody, the anti-CD22 monoclonal antibody inotuzumab ozogamicin (0.8 mg/m^2^) being used in adult ALL, but not yet in infants [7]. Afterwards, we observed a severe tumor lysis syndrome with no measureable leukocytes, indicating that this might be the most effective antibody. Before transplantation, the MRD diagnostics showed only results of 1.0 when blasts could be detected or could not be performed because of absent measurable leukocytes. Although expression of CD19 was identified, we refused to administer the CD19 antibody blinatumomab because of being refractory in first treatment as described above. Shortly after therapy with inotuzumab, allogeneic bone marrow transplantation from an unrelated donor was performed using a special conditioning regimen consisting of thymoglobulin, busulfan, fludarabine, and clofarabine as an additional antileukemic treatment especially in infant ALL with MLL rearrangement [8]. The patient finally recovered completely. A complete molecular remission could be observed in all follow-up bone marrow samples. Currently, 21 months after transplantation, the patient is in a very good physical condition with normal development according to age.

**Table 1:** Immunophenotyping of leukemic cells before and after antibody therapy

Time point	Immunophenotyping
Initial diagnostics	CD33, CD19, CD22, CD79a, CD38, CD10
After 1st gemtuzumab administration	CD38, CD19
After 2nd gemtuzumab and 2nd daratumumab administration	CD22, CD19, CD10

A combination of three different monoclonal antibodies according to the immunophenotype of the leukemic cells can effectively eliminate leukemic cells in chemotherapy-refractory leukemia and serve as bridging to transplant to induce a complete molecular remission afterwards even in infants.
