# Correction to: Molecular remission using three monoclonal antibodies followed by allogeneic bone marrow transplantation in an infant with refractory ALL

**DOI:** 10.1007/s00277-021-04581-1

**Published:** 2021-07-13

**Authors:** Bernd Gruhn, Grit Brodt, Susan Wittig, Thomas Ernst, Jana Ernst

**Affiliations:** 1 Department of Pediatrics, Jena University Hospital, Jena, Germany; 2 Department of Internal Medicine II, Jena University Hospital, Jena, Germany

**Correction to: Annals of Hematology**

10.1007/s00277-020-03957-z

The article “Molecular remission using three monoclonal antibodies followed by allogeneic bone marrow transplantation in an infant with refractory ALL”, written by Bernd Gruhn, Grit Brodt, Susan Wittig, Thomas Ernst and Jana Ernst, was originally published Online First without Open Access. After publication in volume 99, issue 5, page 1133–1134 the author decided to opt for Open Choice and to make the article an Open Access publication. Therefore, the copyright of the article has been changed to © The Author(s) 2021 and the article is forthwith distributed under the terms of the Creative Commons Attribution 4.0 International License, which permits use, sharing, adaptation, distribution and reproduction in any medium or format, as long as you give appropriate credit to the original author(s) and the source, provide a link to the Creative Commons license, and indicate if changes were made. The images or other third party material in this article are included in the article’s Creative Commons license, unless indicated otherwise in a credit line to the material. If material is not included in the article’s Creative Commons license and your intended use is not permitted by statutory regulation or exceeds the permitted use, you will need to obtain permission directly from the copyright holder. To view a copy of this license, visit http://creativecommons.org/licenses/by/4.0.

The original article has been corrected.

